# Single cell RNA sequencing reveals differentiation related genes with drawing implications in predicting prognosis and immunotherapy response in gliomas

**DOI:** 10.1038/s41598-022-05686-x

**Published:** 2022-02-03

**Authors:** Zijian Zhou, JinHong Wei, Zeruo Yang, Yue Bao, Wenbo Jiang, Bin Lu, Weimin Wang, Luo Li

**Affiliations:** 1 Department of Neurosurgery, Qingdao Municipal Hospital, Qingdao University, No.1 Jiaozhou Road, Qingdao, 266011 China; 2 School of Basic Medical Sciences, Southwest Medical University, Luzhou, 646000 China; 3 Center for Quantitative Biology, Academy for Advanced Interdisciplinary Studies, Peking University, Beijing, 100871 China

**Keywords:** Cancer, Computational biology and bioinformatics, Genetics, Immunology, Biomarkers, Health care, Molecular medicine, Oncology, Risk factors

## Abstract

Differentiation states of glioma cells correlated with prognosis and tumor-immune microenvironment (TIME) in patients with gliomas. We aimed to identify differentiation related genes (DRGs) for predicting the prognosis and immunotherapy response in patients with gliomas. We identified three differentiation states and the corresponding DRGs in glioma cells through single-cell transcriptomics analysis. Based on the DRGs, we separated glioma patients into three clusters with distinct clinicopathological features in combination with bulk RNA-seq data. Weighted correlation network analysis, univariate cox regression analysis and least absolute shrinkage and selection operator analysis were involved in the construction of the prognostic model based on DRGs. Distinct clinicopathological characteristics, TIME, immunogenomic patterns and immunotherapy responses were identified across three clusters. A DRG signature composing of 12 genes were identified for predicting the survival of glioma patients and nomogram model integrating the risk score and multi-clinicopathological factors were constructed for clinical practice. Patients in high-risk group tended to get shorter overall survival and better response to immune checkpoint blockage therapy. We obtained 9 candidate drugs through comprehensive analysis of the differentially expressed genes between the low and high-risk groups in the model. Our findings indicated that the risk score may not only contribute to the determination of prognosis but also facilitate in the prediction of immunotherapy response in glioma patients.

## Introduction

Gliomas are the most prevalent primary tumors originated from the central nervous system and are among the most devastating forms of cancer^1^. They vary in terms of pathological characteristics from benign to malignant and are divided into four grades (I to IV) by the World Health Organization (WHO)^2,3^. Due to the high heterogeneity of gliomas, the traditional classification is not satisfactory for predicting the prognosis even for patients with the same diagnosis. A more efficient strategy for clinicians to predict the prognosis of glioma patients is urgently needed. While there have been a large number of gene signatures serving as prognostic models, the predictive performance remained to be further improved concerning the accuracy and clinical practice^4–10^. Moreover, little prognostic models have been established which could be applicable to both glioblastoma (GBM) and low-grade glioma (LGG). In addition, the prognosis for patients with gliomas can be dismal combing radiotherapy and chemotherapy after surgical resection of the tumor^11,12^. As a novel therapeutic strategy, immunotherapy has been extensively investigated in more and more cancers and durable responses to immunotherapy in many other forms of cancers have drawn increasing attention of the study of immunotherapy in gliomas^13^. However, only the minority of patients with gliomas got effective immune responses in clinical trials due to lack in precise selection with predictive biomarkers at this time^14^. There is an urgent need for an efficient classification or biological predictors with implications in discriminating the prognosis and immunotherapy responses of glioma patients.

Glioma is driven by GSCs which mainly account for the failure of current treatment strategies against malignant glioma. An increasing number of studies have shed light on the existence of glioma stem cells (GSCs), which are capable of self-renewal and responsible for the origin of tumors. Considering the pluripotency of GSCs, they could differentiate into multiple cell subgroups^15^, leading to high of heterogeneity of cell differentiation states^16^. Most of malignant tumors such as glioblastoma are characterized by incomplete differentiation and differentiated glioma cells tend to get benign characteristics^17^. It is widely accepted that the differentiation states correlated with the maintenance and progression of malignant tumors so as to influence the fate and prognosis of patients. In addition, the differentiation states of GSCs are reported to be potentially associated with the drug resistance^18^. The mechanisms underlying cell differentiation remain unclear and the determination of differentiation related genes (DRGs) involved in GSCs with respect to astrocytes could contribute to the identification of new biomarkers and treatment strategies against gliomas. Previous studies have demonstrated that the expression of four neurodevelopmental transcription factors, including *SALL2*, *SOX2*, *OLIG2*, and *POU3F2*, were associated with the differentiation of GSCs, in which *FOSL1* expression could promote cell differentiation by repressing the four transcription factors^19^. King et al. revealed that *ASIC1a* expression negatively correlated with glioma grading and played an important role in directing GSCs toward differentiation^20^. Differentiation therapy which tries to induce cancer stem cells into more differentiated and less malignant states was highlighted in recent years^21^. The exact molecular mechanisms underlying this novel treatment strategy have not been sufficiently defined yet. We try to appeal the implications in finding the underlying therapeutic targets for differentiation therapy through comprehensive analysis of DRGs in this study. The multiple factors in the tumor-immune microenvironment play a great role in the differentiation of cancer stem cells (CSCs) and drive progression of tumors^22–24^. Furthermore, it has been well-established that single-cell transcriptomics analysis provides a new approach for investigating the heterogeneity of tumors at cellular resolution^25^. To date, there are rare papers focusing on the construction of gene signatures based on DRGs for predicting prognosis and immunotherapy responses. In addition, considering that the effect of conventional chemotherapy against gliomas is not satisfactory due to drug resistance, comprehensive analysis of DRGs facilitates in the development of novel therapeutic gene targets for predicting candidate drugs^11^.

In this study, we attended to explore the multiple differentiation states of glioma cells through analysis of single cell RNA sequencing (scRNA-seq) of gliomas to identify DRGs for predicting prognosis, immunotherapy response and candidate targeted drugs combining with bulk RNA-seq data.

## Results

### Quality control and filtration of scRNA-seq data

**Figure 1:**
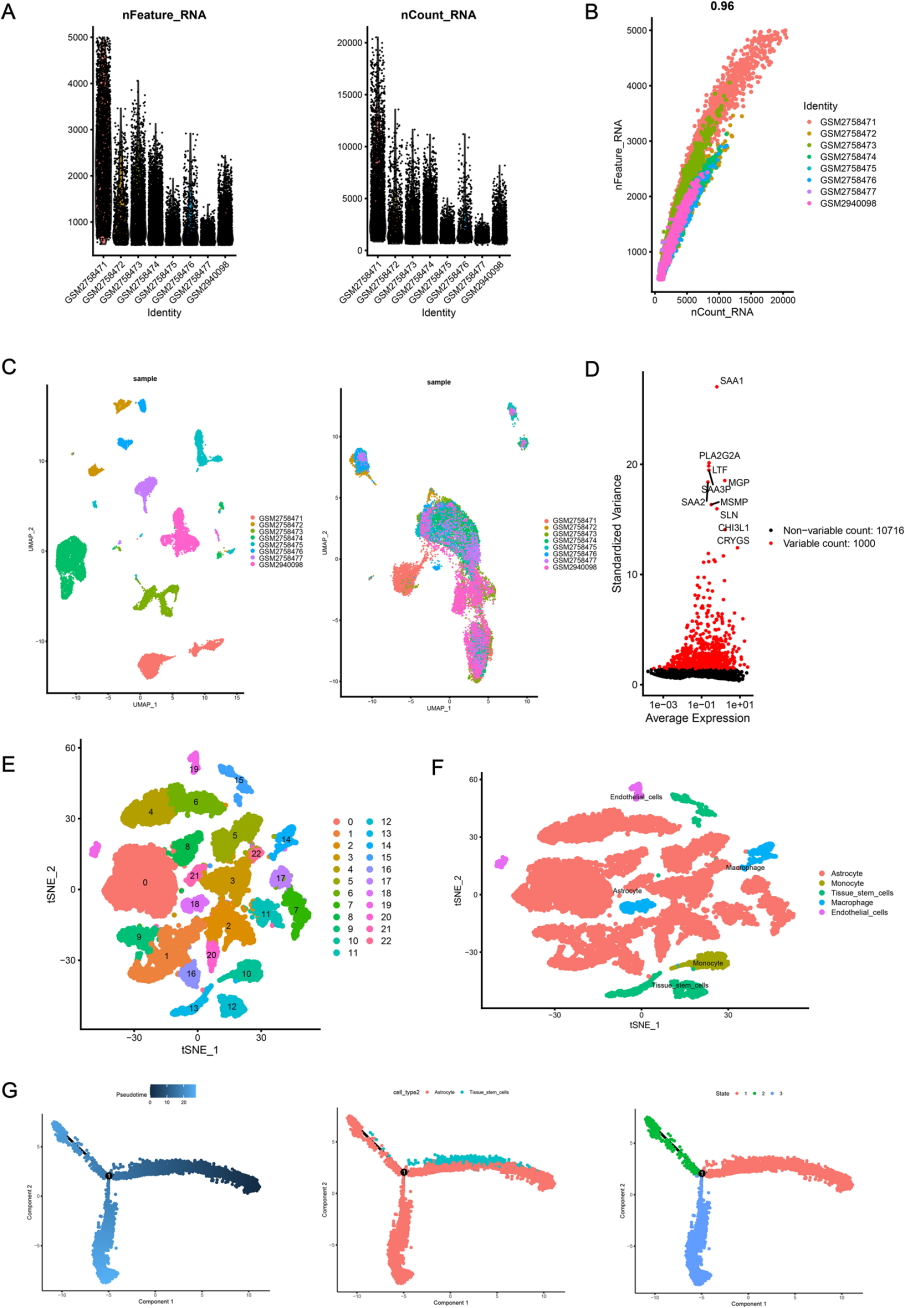
Clustering and differentiation trajectory analysis for cells based on single-cell RNA-seq data. (**A**) Violin plot displaying the result of quality control and fitration of the scRNA-seq dada. (**B**) Scatter plot showing the correlation between sequencing depth and the number of detected genes. (**C**) UMAP analysis showing the result of batch effect correction in which the left panel represented the result before batch effect correction and the right panel represented the result after batch effect correction. (**D**) Scatter plot showing 1000 highly variable genes across the total samples with top 10 genes annotated. (**E**) Scatter plot showing 23 cell clusters processed by the tSNE algorithm based on 10 PCs. (**F**) Scatter plot showing the cells annotated with cell types. (**G**) Differentiation trajectory analysis identifying 3 branches of astrocyte and tissue stem cells with distinct differentiation state and pseudotime. UMAP: Uniform Manifold Approximation and Projection, PCA: principal component analysis, tSNE: t-distributed stochastic neighbor embedding.

After quality control, filtration and batch effect correction of the scRNA-seq data, 15,853 cells were acquired from GSE103224 dataset (Fig. 1A). There was significant positive correlation between sequencing depth and the number of detected genes (R = 0.96, Fig. 1B). The result of batch effect correction was shown by UMAP (Uniform Manifold Approximation and Projection) analysis in Fig. 1C. A total of 11,773 genes were detected and 1000 highly variable genes were depicted in Fig. 1D.

### Clustering and differentiation trajectory analysis for glioma cells

10 PCs (principal components) with statistical significance were determined for clustering analysis of the cells (Fig. S1A). Based on tSNE algorithm, 15,853 cells were separated into 23 clusters and the expression patterns of top 10 differentially expressed marker genes for each cluster were displayed in the heatmap (Fig. S1B). The cells distributed in 23 clusters were annotated with cell types according to marker genes (Fig. 1E,F), in which cluster 14 and 18 were macrophages, cluster 10 were monocytes, cluster 12, 13 and 15 tended to be closely related to stem cells, cluster 19 tended to be related to epithelial cells, and the remaining clusters tended to be close to astrocytes. Pseudotime and differentiation trajectory analysis was carried out for 12,456 astrocyte and stem cells, and 3 branches of cells with distinct differentiation patterns were identified. Cells distributed in state 1 were assumed to be the initial type of cells and then differentiated into other states (Fig. 1G).

### Functional annotation for three differentiation states

According to the marker genes for specific differentiation states, KEGG (Kyoto Encyclopedia of Genes and Genomes) pathway analysis was carried out to reveal the enriched potential molecular mechanisms. Marker genes for differentiation state 1 were correlated with cellular senescence, glioma and p53 signaling pathway. Marker genes in state 2 tended to be related to cellular senescence, glioma, cell cycle, HIF − 1 signaling pathway and apoptosis (Fig. S2).

### Classification for glioma patients based on DRGs

**Figure 2:**
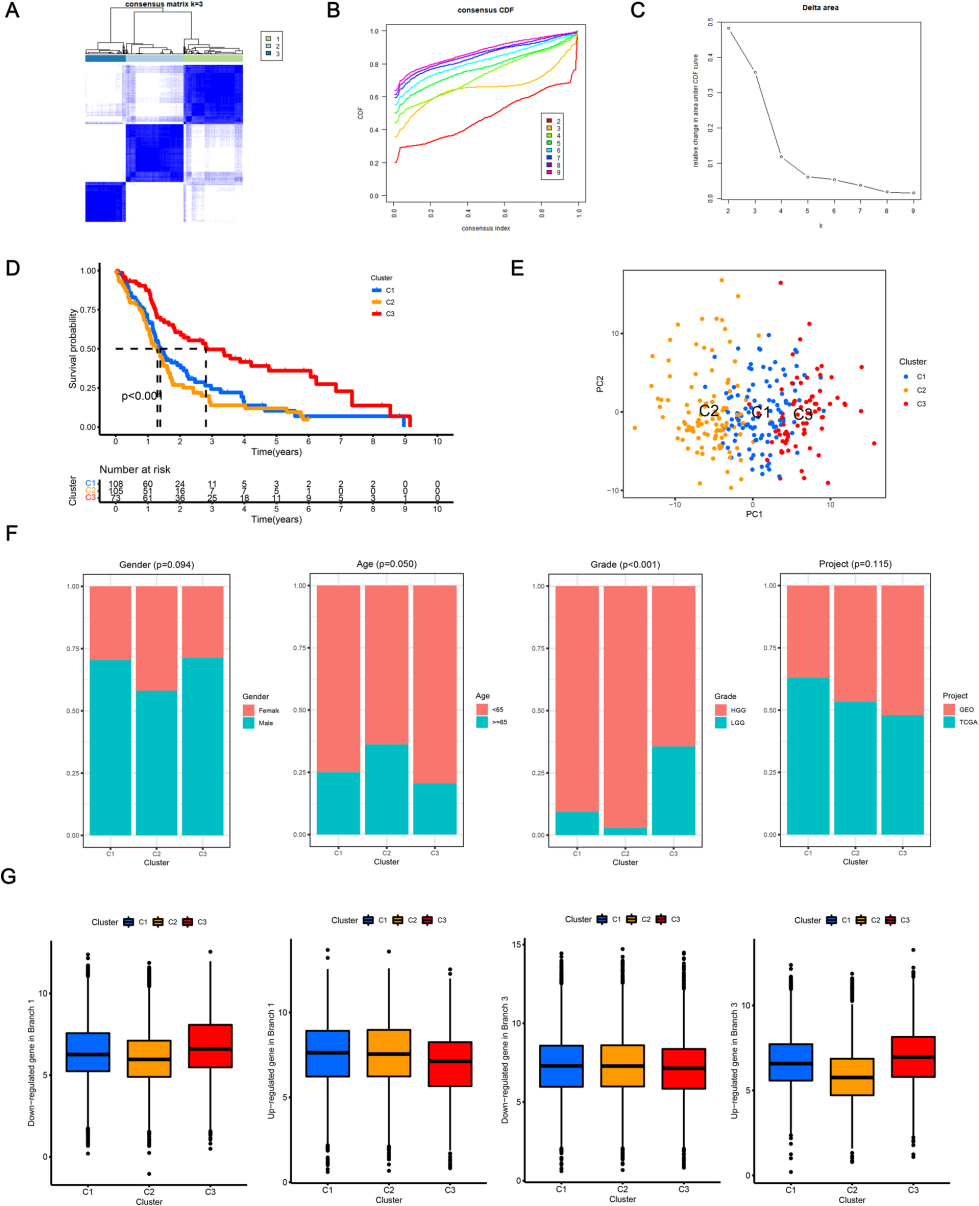
Classification for glioma patients based on DRGs. (**A**–**C**) The results of consensus clustering analysis for glioma patients based on DRGs. (**D**) Overall survival analysis between patients in the three clusters. (**E**) Scatter plot demonstrating the result of PCA for the classification of glioma patients based on DRGs. (**F**) Comparisons of clinicopathological features across the three clusters. (**G**) The up/down-regulated DRGs in differentiation state 1 and 3 showed similar expression patterns in C2 and C3 clusters, respectively. DRGs: differentiation related genes, PCA: principal component analysis.

**Figure 3:**
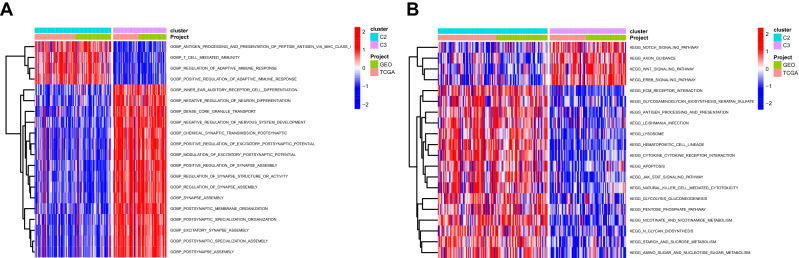
Differentially analysis of enriched functions between C2 and C3. (**A**) Heatmap illustrating the differentially enriched biological process between C2 and C3 clusters. (**B**) Heatmap illustrating the differentially enriched KEGG pathways between C2 and C3 clusters. KEGG: Kyoto Encyclopedia of Genes and Genomes.

A total of 833 marker genes for three cell differentiation states were regarded as DRGs. Through consensus clustering analysis, 3 molecular clusters with distinct overall survival (*p* < 0.001) were identified based on the expression profiles of DRGs in the merged data set including patients from TCGA-GBM data, GSE4271 and GSE43378 dataset (Fig. 2A–C). Patients in C3 tended to have the best prognosis, whereas patients in C2 exhibited worse overall survival (Fig. 2D). The result of PCA (principal component analysis) demonstrated that the classification could significantly distinguish glioma samples (Fig. 2E). In addition, the classification of gliomas based on highly variable genes identified in scRNA-seq data was performed via consensus clustering analysis. As depicted in Fig. S3A, patients were appropriately classified into two clusters. Kaplan–Meier survival analysis indicated that the prognosis between the two clusters exhibited no significant difference (Fig. S3B) implying that the classification based on DRGs showed an advantage compared to highly variable genes with drawing implications in discriminating prognosis in gliomas. As shown in Fig. 2F, the pathological grade for patients of C3 tended to be lower than those of other clusters (*p* < 0.001), which was consistent with the results of overall survival analysis. The up/down-regulated DRGs in differentiation state 1 presented similar trend in samples of C2, and the expression patterns of up/down-regulated DRGs in differentiation state 3 showed similar trend in samples of C3, which implying that glioma cells of C2 were mainly in differentiation state 1 which represented a less differentiated state and cells of C3 were mainly in differentiation state 3 which represented a more differentiated state (Fig. 2G). As shown in Fig. 3A,B, the results for differential analysis of enriched biological process and pathways between C2 and C3 clusters demonstrated that, compared with C3, gliomas of C2 mainly correlated with immune responses (e.g., T cell mediated immunity and positive regulation of adaptive immune response) and tumorigenesis (e.g., ECM receptor interaction and apoptosis).

### Identification of distinct tumor-immune microenvironment (TIME), immunogenomic patterns and immunotherapy response across different clusters

**Figure 4:**
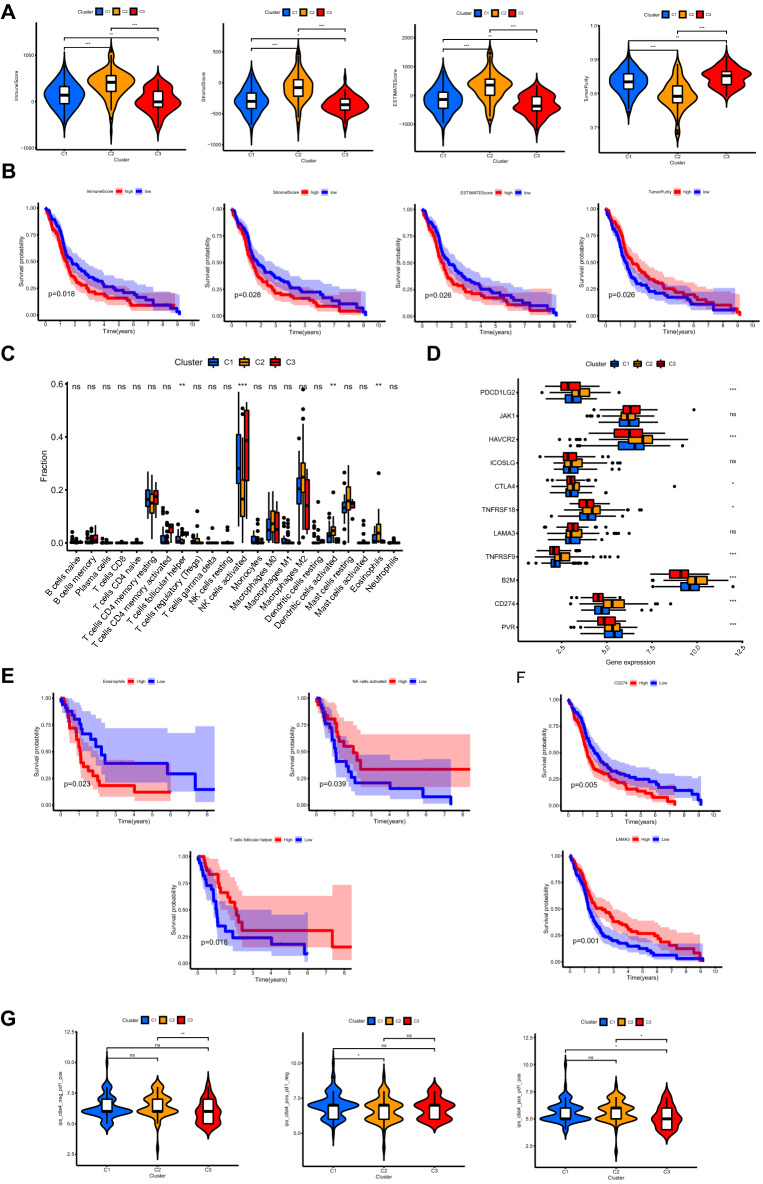
Identification of distinct TIME, immunogenomic patterns and immunotherapy response across different clusters. (**A**) Comparisons of immune score, stromal score, ESTIMATE score and tumor purity. (**B**) Kaplan–Meier survival analysis for patients with distinct TIME. (**C**) Comparisons of the abundances of infiltrating immune cells. (**D**) Comparisons of the expression levels of immune checkpoints. (**E**) Kaplan–Meier survival analysis for patients with distinct abundance of infiltrating immune cells. (**F**) Kaplan–Meier survival analysis for patients with distinct expression levels of immune checkpoints. (**G**) Comparisons of immunotherapy scores. ‘ns’ means no significance, * means *P* < 0.05, ** means *P* < 0.01, and ***means *P* < 0.001. TIME: tumor-immune microenvironment.

The immune, stromal, and ESTIMATE scores for samples in cluster C2 were the highest and the tumor purity was the lowest in cluster C2 (all *p* < 0.001), indicating the highest abundance of immune and stromal cells and the lowest tumor purity in C2 (Fig. 4A). Conversely, the results demonstrated the lowest abundance of immune and stromal cells and the highest tumor purity in C3. Kaplan–Meier survival analysis for patients with distinct TIME was used to investigate the correlation of TIME and overall survival (Fig. 4B). Patients with lower immune, stromal, and ESTIMATE scores and higher tumor purity tended to have a better survival. Moreover, follicular helper T cells and activated NK cells were significantly more abundant in C3 tumors. However, eosinophils and activated dendritic cells were more abundant in C2 tumors (Fig. 4C). The expression levels of immune checkpoints including *PDCD1LG2, HAVCR2, CTLA4, TNFRSF18, TNFRSF9, B2M,* and *CD274* were the highest in C2 tumors (Fig. 4D). Patients with higher abundance of infiltrating activated NK cells or follicular helper T cells presented better overall survival. In contrast, patients with higher abundance of infiltrating eosinophils presented worse overall survival (Fig. 4E). Moreover, patients with higher expression levels of *CD274* tended to have worse prognosis and patients with higher expression levels of *LAMA3* tended to have better prognosis (Fig. 4F). As shown in Fig. 4G, the scores for anti-PD1 immunotherapy of C2 were significantly higher than those in C3, indicating that patients in C2 tended to benefit from anti-PD1 therapy (*p* < 0.01). As for anti-CTLA4 immunotherapy, patients in C1 tended to get effective response compared to C2. Patients in C3 were less likely to get response than those in C2 or C1 when both anti-PD1 and anti-CTLA4 were applied.

### Construction and validation of prognostic model based on DRGs

**Figure 5:**
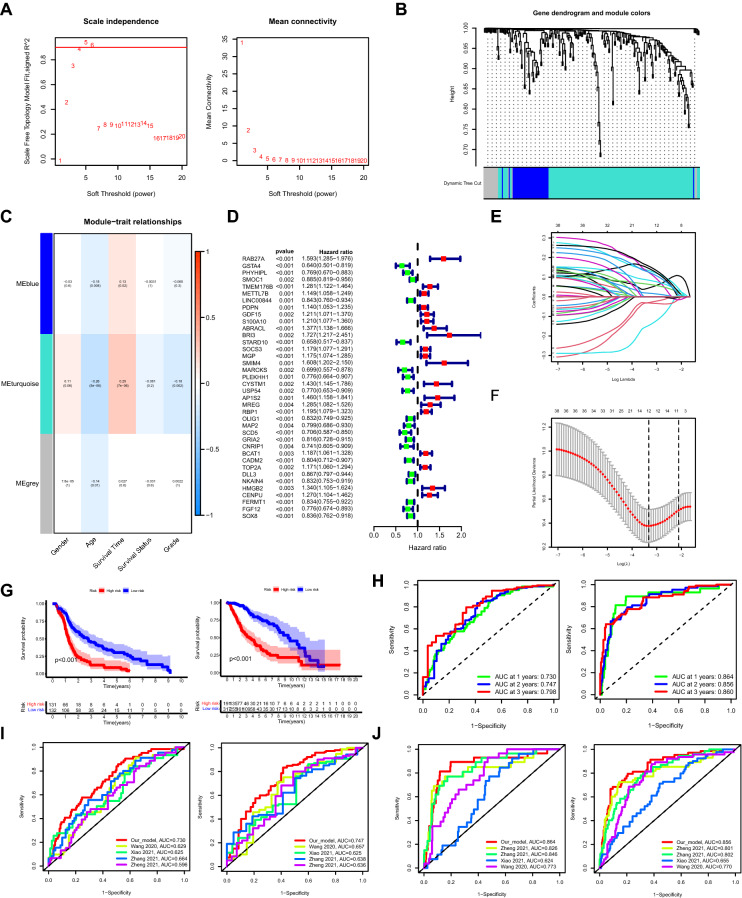
Construction of prognostic model based on DRGs. (**A**,**B**) Three modules were identified with the optimal soft threshold = 4 based on WGCNA. (**C**) The result of correlation analysis between modules and clinicopathological features. (**D**) Forest plot displaying the result of univariate cox analysis of DRGs. (**E**) The coefficient profile plot against the log(lambda) sequence in the LASSO model. (**F**) Optimal parameter (lambda) selection in the LASSO model. (**G**) Kaplan–Meier analysis between the low-risk group and the high-risk group in the training (left panel) and validation (right panel) cohorts, respectively. (**H**) The ROC curves for predicting 1-year, 2-year and 3-year overall survival in the training (left panel) and validation (right panel) cohorts, respectively. (**I**) The ROC curves showing the comparison between our prognostic model and other recently established models in the training cohort for predicting 1-year (left panel) and 2-year (right panel) overall survival, respectively. (**J**) Similar results in the validation cohort. DRGs: differentiation related genes, WGCNA: weighted correlation network analysis, LASSO: least absolute shrinkage and selection operator, ROC: receiver operating characteristic.

**Figure 6:**
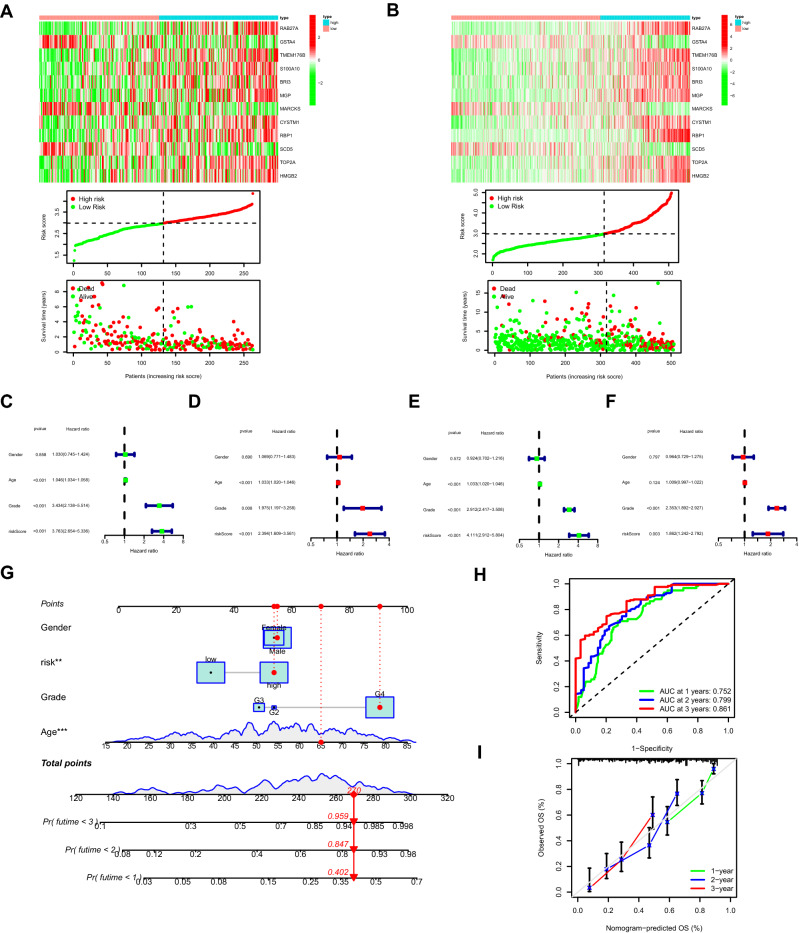
Construction and evaluation of the nomogram. (**A**) Heatmap showing the expression patterns of prognostic genes in the model, the curve of risk scores and scatter plot of survival states in the training cohort. (**B**) Verification of the above results using the validation cohort. (**C**) Forest plot showing the results of univariate cox regression analysis of the risk score and clinicopathological features in the training cohort. (**D**) Forest plot showing the results of multivariate cox regression analysis of the risk score and clinicopathological features in the training cohort. (**E**,**F**) Verification of the above results using the validation cohort. (**G**) Nomogram integrating risk, age, gender, grade as variables with predictions of survival at 1, 2, and 3 years using the training cohort. (**H**) The ROC curves of the nomogram for predicting 1-year, 2-year and 3-year overall survival in the training cohort. (**I**) The calibration curves evaluating the nomogram.

Through WGCNA (weighted correlation network analysis) integrating clinicopathological data and the expression profiles of DRGs in the merged data, three modules were screened out with the optimal soft threshold = 4 (Fig. 5A,B), in which the turquoise module significantly correlated with both the survival time of glioma patients and the grade of tumors (Fig. 5C). A total of 163 genes in the turquoise module were selected to be enrolled in the univariate cox analysis. Afterwards, a total of 38 DRGs with prognostic values were identified (Fig. 5D). Finally, a prognostic model consisting of 12 genes were constructed through the least absolute shrinkage and selection operator (LASSO) regression algorithm (Fig. 5E,F). The 12 genes involved in the prognostic model with corresponding coefficients were listed in Table 1. Patients were divided into high-risk group and low-risk group with the cut off of the median risk score. As shown in Fig. 5G, the overall survival for patients in the low-risk group was significantly higher than those in the high-risk group either in the training or validation cohort (*p* < 0.001). In order to evaluate the performance of the prognostic model in subgroups with different clinicopathological features, patients were divided into different subgroups according to gender and grade. Similar results were obtained in which patients in the low-risk group tended to get better survival compared with those in the high-risk group (Fig. S4A). Moreover, the areas under the receiver operating characteristic (ROC) curves (AUC) for predicting 1-year, 2-year and 3-year overall survival were 0.730, 0.747, 0.798, respectively, in the training cohort, and 0.864, 0.856, 0.860, respectively, in the validation cohort (Fig. 5H). Four recently published glioma related prognostic models were compared with our model. In the training cohort, the AUC of our model for predicting 1-year and 2-year overall survival were higher than other models (Fig. 5I). In addition, the accuracy of our model was also better than other glioma related models in the validation cohort^4,6,7,26^ (Fig. 5J). As demonstrated in Fig. 6A,B, the expression levels of *RAB27A, TMEM176B, S100A10, BRI3, MGP, CYSTM1, RBP1, TOP2A, and HMGB2* increased with the increasing of the risk scores, indicating these genes acted as risk genes. Alternatively, *GSTA4*, *SCD5*, and *MARCKS* acted as protective genes. The survival time and survival rate decreased with the increasing of the risk scores. The expression levels of the 12 prognostic genes in the model significantly differed between patients of the low and high-risk groups (Fig. S4B,C). In the training cohort, the result of univariate cox analysis revealed that age, grade and risk score were significantly associated with the prognosis of glioma patients (Fig. 6C). The results of multivariate cox analysis indicated that age, grade and risk score can be independent factors for predicting the prognosis of glioma patients (Fig. 6D). Similar results were obtained in the validation cohort (Fig. 6E,F). An independent cohort from CGGA database (data set ID: mRNAseq_325) was employed to further verify the performance of the model (Fig. S5). The corresponding clinical information was shown in Supplementary Table 6.

**Table 1:** Prognostic genes involved in our model.

Gene	Coefficients
RAB27A	0.0447783869529816
GSTA4	− 0.0294365949555612
TMEM176B	0.133932069262102
S100A10	0.0183174232803931
BRI3	0.124908133380746
MGP	0.0636231985984361
MARCKS	− 0.146500790307173
CYSTM1	0.145004134475847
RBP1	0.0847420516917888
SCD5	− 0.0316244289214241
TOP2A	0.156547345519961
HMGB2	0.125638780008089

### Establishment and evaluation of nomogram

Nomogram integrating age, gender, grade and risk group was constructed for predicting the survival of glioma patients at 1, 2 and 3 years based in the training cohort (Fig. 6G). The AUC for predicting 1-year, 2-year and 3-year overall survival were 0.752, 0.799 and 0.861, respectively (Fig. 6H). Calibration curves for predicting 1-year, 2-year and 3-year overall survival were close to the actual observed values (Fig. 6I).

### Identification of immunotherapy response for patients of high and low-risk groups

**Figure 7:**
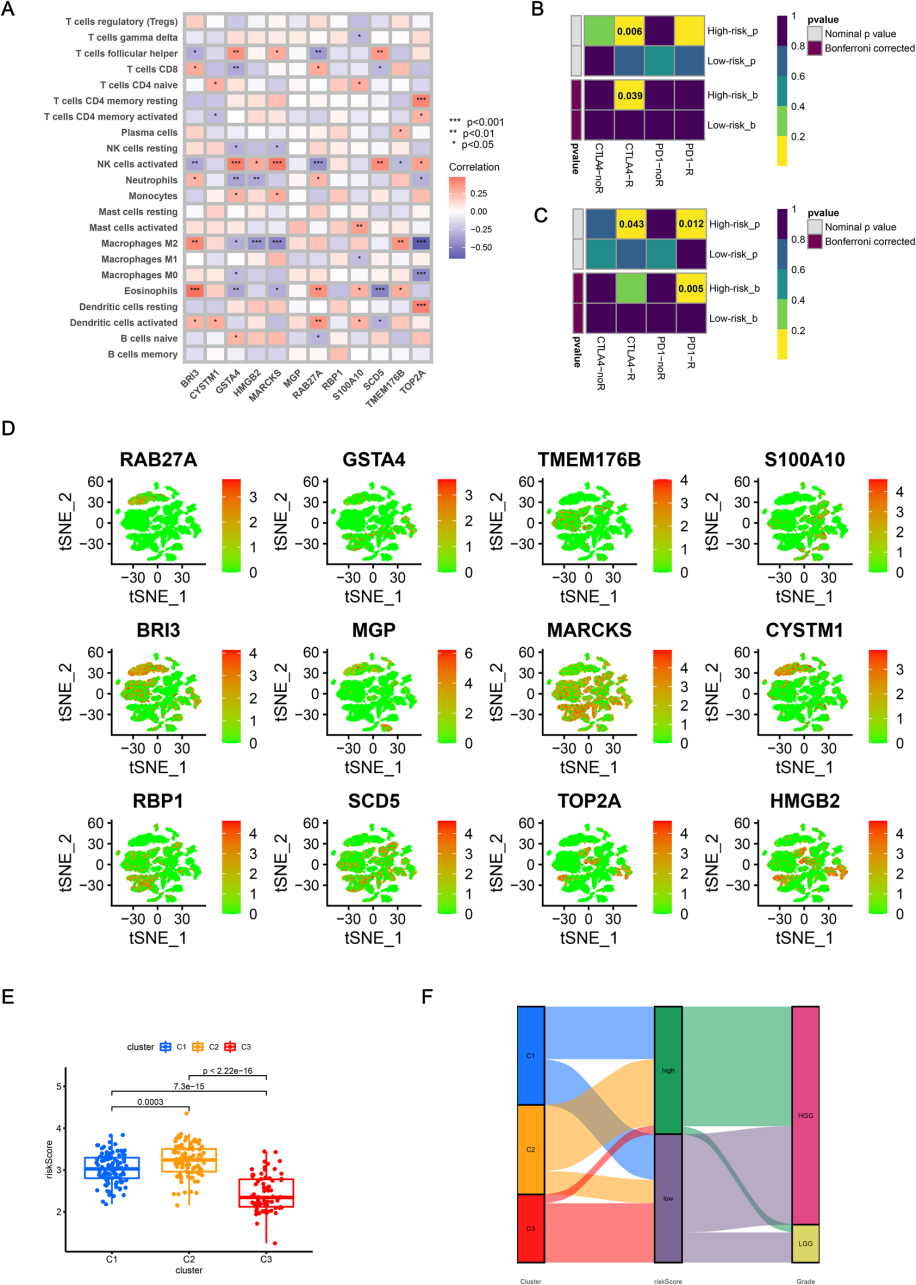
(**A**) Heatmap showing the correlation between the expression levels of 12 prognostic genes in our model and the abundance of infiltrating immune cells. (**B**) Unsupervised subclass mapping analysis of the response to immunotherapy between high-risk group and low-risk group in the training cohort. (**C**) Verification of the above results using the validation cohort. (**D**) The expression patterns of the 12 prognostic genes in the subgroups of the single-cell RNA-seq data. (**E**) Comparison of the risk scores across the three clusters. (**F**) Alluvial diagram showing the distribution of three clusters in different risk groups and grades.

The correlation between the expression levels of 12 prognostic genes in our model and the abundance of infiltrating immune cells was identified in Fig. 7A. Among them, the expression levels of *GSTA4, HMGB2, MARCKS,* and *TOP2A* positively correlated with the infiltration of activated NK cells and negatively correlated with the infiltration of M2 macrophages, indicating the potential correlation between the expression patterns of prognostic genes and TIME. Furthermore, unsupervised subclass mapping analysis of immunotherapy response between the high-risk group and low-risk groups was carried out (Fig. 7B,C). In the training cohort, patients in the high-risk group tended to get better responses to anti-CTLA4 immunotherapy than those in the low-risk group (*p* = 0.039). As for the validation cohort, patients in the high-risk group were more sensitive to either anti-PD1 therapy (*p* = 0.005, respectively).

### The expression patterns of the 12 prognostic genes in the subgroups of scRNA-seq data

As shown in Fig. 7D, the expression patterns of the 12 prognostic genes were diverse among different subgroups of cells. Compared with other prognostic genes, *MARCKS* were widely expressed in most of the cells. *BRI3, RBP, SCD5,* and *HMGB2* were highly expressed in astrocytes.

### Identification of the risk scores for glioma patients in three clusters

The risk scores for patients in C2 were significantly higher compared with other clusters (Fig. 7E) which was consistent with the previous results. As shown in Fig. 7F, most of the patients in C2 distributed in high-risk group and most of the patients in C3 distributed in low-risk group, which was in agreement with the above results. Moreover, the correlation between prognostic DRGs and the corresponding transcriptional factors was demonstrated in Fig. S6.

### Prediction of candidate targeted drugs

A total of 479 differentially expressed genes (DEGs) between the low and high-risk groups were screened out for further analysis in Connectivity Map (CMap). Nine candidate targeted drugs with potential therapeutic values which can reverse the expression patterns of patients in the high-risk group were identified (Table 2).

**Table 2:** The candidate small molecules with highly significant correlations in the results of CMap.

cMap name	Enrichment	*p*-value
Y-27632	− 0.928	0.01076
5230742	− 0.925	0.01147
guanethidine	− 0.924	0.00074
AH-23848	− 0.913	0.00114
puromycin	− 0.885	0.00038
NU-1025	− 0.865	0.03634
oxybutynin	− 0.851	0.00093
benzthiazide	− 0.835	0.00135
clofilium tosylate	− 0.831	0.00959

## Discussion

In this study, we identified three cell differentiation states in glioma tissues through analysis of single-cell RNA-seq data from GEO database and the marker genes for specific cell states were subsequently merged as DRGs, based on which we separated glioma patients into three clusters with distinct clinicopathological features based on the bulk RNA-seq data from TCGA and GEO database. Distinct TIME, immunogenomic patterns and immunotherapy responses were identified across three clusters. A prognostic model composing of 12 DRGs was established to predict the prognosis of glioma patients and nomogram model integrating the risk group and multi-clinicopathological factors were constructed for clinical practice. We obtained nine candidate targeted drugs through comprehensive analysis of the DEGs between the low and high-risk groups in the model.

Single-cell RNA-seq technology provided a new method to explore the intratumoral heterogeneity through analyzing the transcriptional characteristics at the resolution of single cells^27^. The single-cell RNA-seq data composing of high-grade gliomas (HGGs) were applied in this study. Consistent with previous studies, while the transformed cells in HGG resemble glia^28,29^, we identified a rare subpopulation of GSCs which gave the possibility to explore the multiple cell differentiation states in glioma cells. Cells in gliomas differentiated from GSCs exhibited distinct marker phenotypes, morphologies, and migratory properties^30^. Given that gliomas represent a heterogeneous group of tumors originating from astrocytes and oligodendrocytes, we selected astrocytes and tissue stem cells for the differentiation trajectory analysis based on the annotation of all cells involved in the glioma tissues. We identified three clusters in glioma patients with distinct clinicopathological features based on the expression patterns of DRGs. In addition, the correspondence between the clusters and the differentiation states suggested that the prognosis and immunotherapy responses correlated with the cell differentiation states in glioma patients. For example, gliomas of patients in C2, which had worse overall survival and higher risk scores, mainly distributed in less differentiated state (state 1) and C3 which had better prognosis and lower risk scores tended to distributed in more differentiated state (state 3), implying that less differentiated state correlated with poor prognosis which was consistent with the well-established theory^21^. The investigation focusing on the identification of DRGs promoted a better understanding of the prognosis and treatment of gliomas. Previous studies demonstrated that cell signaling pathways and transcriptional cascades involved in the progress of differentiation have been implicated in the oncogenesis and progression of malignant tumors. Differentiation therapy, in which cancer cells were induced by transformative signaling events and then directed towards more differentiated and less malignant states, shed light on the treatment of malignant tumors including those occurring in the brain and central nervous system^31^. While significant progress regarding of differentiation therapy in gliomas has been made, the specific molecular mechanisms and therapeutic targets remains unclear^32–34^. In this study, we screened out DRGs with prognostic values and the corresponding transcriptional factors to provide promising candidate targets for prospective differentiation therapy. On the other hand, Wang et al. identified an immune-related signature indicating the dedifferentiation of thyroid cells, which suggested a correlation between differentiation states and TIME^5,35^. To date, there has been rare related study concerning the correlation between tumor differentiation status and TIME in gliomas. In our research, the tumor microenvironment for gliomas of C2 exhibited more infiltrating immune cells and lower tumor purity. Tumor-associated macrophages, presenting an M2-like polarization, were the most abundant leucocyte subset in cancers and exhibited anti-inflammatory effects in TIME, which promoted tumorigenesis and progression through regulating anti-tumor immune processes^36^. Consistent with the previous studies, we found that two favorable DRGs in our prognostic model including *GSTA4* and *MARCKS* negatively correlated with the infiltration of M2 macrophages while two unfavorable DRGs including *BRI3* and *TMEM176B* positively correlated with the infiltration of M2 macrophages. However, the unfavorable DRGs including *HMGB2* and *TOP2A* presented adverse trend in our study which might need further investigation. Moreover, patients in C2 were sensitive to immune checkpoint blockage therapy which was consistent with the results that patients in the high-risk group tended to respond to anti-PD1 or anti-CTLA4 immunotherapy. All these findings raised the possibility of predicting TIME, immunogenicity and immunotherapy responses based on the expression patterns of DRGs in glioma patients.

Compared to other prognostic models with respect to gliomas, the values of AUC for our model were higher which indicated higher accuracy for predicting the prognosis of glioma patients. Moreover, we extended our research to patients with low-grade glioma in which the performance of the prognostic model was powerful as well. Unsupervised subclass mapping analysis suggested that glioma patients with higher risk scores tended to have a better response to immune checkpoint blockage therapy than those with lower risk scores regardless of the weak statistical significance, implying that the risk score can also serve as an indicator indicative of the response to immunotherapy. On the other hand, we found nine candidate drugs with potential therapeutic efficacy based on the DEGs between low and high-risk groups by using bioinformatics analysis, thus contributing to the drug investigation in the future.

There were some drawbacks in the current study. Firstly, due to the lack of single-cell RNA-seq data of LGG patients in the GEO database, the DRGs were mainly derived from scRNA-seq data of HGG patients. Moreover, the proportion of LGG patients in the training cohort was lower compared to HGG patients. However, the performance of our model was further evaluated and verified in the validation cohort which was composed of LGG samples. Secondly, the specific mechanism of the DRGs in TIME remained unclear. Thirdly, Molecular and cellular biological experiments were needed to further verify our findings.

## Conclusion

Our prognostic model based on differentiation related gene signature has a potential value in predicting prognosis, immunotherapy responses in glioma patients. Thus, the risk score can serve as a powerful biomarker for prognosis and immunotherapy response. Besides, we screened out candidate drugs based on the DEGs between the low and high-risk groups for guiding the direction of future drug exploration.

## Methods

### Acquisition of scRNA-seq data and bulk RNA-seq data

We declare that all the data supporting the findings of this study are available in the TCGA database (https://portal.gdc.cancer.gov/), GEO database (https://www.ncbi.nlm.nih. gov/geo/) and CGGA database (Chinese Glioma Genome Atlas, http://cgga.org.cn/index.jsp). We confirm that all methods used in this study are performed in accordance with the relevant guidelines and regulations. The scRNA-seq data consisting of 23,793 glioma cells from 8 patients were obtained from GSE103224 dataset^37^ in the GEO database. The corresponding clinicopathological features were listed in Supplementary Table 1. ‘Seurat’ and ‘Monocle’ packages in R software (version 4.1.1) were used in the preprocessing of scRNA-seq data^38^. The content of mitochondrial genes was calculated through the PercentageFeatureSet function. Genes detected in < 3 cells were excluded and cells with < 500 or > 5000 detected genes and ≥ 2.5% mitochondrial expressed genes were excluded, after which 15,853 cells were selected for further analysis. Harmony algorithm was employed to correct the batch effects of the scRNA-seq data by using ‘harmony’ R package, followed which UMAP (Uniform Manifold Approximation and Projection) was utilized to assess and visualize the results^39^. ScRNA-seq data were normalized by the ‘LogNormalize’ method, and the top 1000 highly variable genes were identified by the ‘vst’ selection method. Bulk RNA-seq data of patients with GBM and low-grade glioma (LGG) were acquired from TCGA (http://cancergenome.nih.gov/) database. The corresponding clinicopathological information was acquired from cBioPortal database (https://www.cbioportal.org/). GSE4271 and GSE43378 data sets were obtained from the GEO database. The corresponding clinicopathological information for the four cohorts was presented in Supplementary Tables 2–5. Ethics committee approval for our study was not required because the data were obtained from publicly available databases.

### Processing of the scRNA-seq data

Firstly, PCA was utilized for dimension reduction of the glioma cells^40^. The top 10 principal components (PCs) with significant values were selected for clustering by using the t-distributed stochastic neighbor embedding (tSNE) algorithm^41^. Marker genes with Log2 [fold change (FC)] > 1 and adjusted *p* values < 0.05 in each cluster were identified using the ‘limma’ package. ‘SingleR’ package was employed as an automatic annotation method for scRNA-seq data in this study^42^. Human primary cell atlas data involved in ‘celldex’ package were used as reference data^43^.

### Pseudotime and trajectory analysis

Pseudotime and trajectory analysis were conducted for astrocyte and tissue stem cells by using ‘Monocle’ package. Cells distributed in the same branch were considered to be in the same differentiation state. DEGs in cells with distinct differentiation states were determined by |log2 (FC)|> 1 and adjusted *p* values < 0.05, which were defined as DRGs. The KEGG pathway analysis for DRGs was carried out by ‘clusterProfiler’, ‘org.Hs.eg.db’, ‘enrichplot’ and ‘ggplot2’ packages^44–46^. The KEGG pathway related data were publicly available on the website (https://www.kegg.jp/kegg/kegg1.html).

### Classification for glioma patients based on DRGs

The bulk RNA-seq data for GBM samples were transformed to transcripts per million (TPM) values and log2-scale transferred for normalization and subsequently merged with transcriptional data from GSE4271 and GSE43378 dataset. The merged data were corrected batch effect by using R package ‘sva’. DRGs expression patterns were extracted for further analysis. Unsupervised consensus clustering for glioma patients was carried out by using ‘ConsensusClusterPlus’ package based on expression patterns of DRGs^47^. 50 iterations with maxK = 9 were utilized for stable classification. Kaplan–Meier analysis was employed to compare the overall survival of patients across different clusters. The distribution of clinicopathological features in each cluster was visualized by ‘ggplot2’ package. The expression levels of the marker genes for specific differentiation states were explored across different clusters. PCA was conducted to evaluate the results of clustering. Gene set variation analysis (GSVA) was utilized for differential analysis of enriched molecular functions and pathways between different clusters by using ‘limma’, ‘GSVA’ and ‘GSEABase’ R packages. The GO biological process and KEGG pathways with |log2FC|> 0.1 and FDR < 0.05 were considered significantly enriched molecular functions and pathways between different clusters. The KEGG and ontology gene sets (c5.go.bp.v7.4.symbols.gmt, c2.cp.kegg.v7.4.symbols.gmt) used for GSVA were downloaded from GSEA database (https://www.gsea-msigdb.org/gsea/index.jsp).

### Identification of TIME and immune patterns of patients across different clusters

The immune score, stromal score, ESTIMATE score and tumor purity of each sample were calculated through ‘ESTIMATE’ package. The abundance of infiltrating immune cells of each sample was determined by using CIBERSORT method and ‘limma’ package. The expression levels of immune checkpoints^48–55^ in glioma patients across different clusters were also evaluated for differential analysis. Kaplan–Meier analysis was used to investigate the correlation between TIME and overall survival. The immunotherapy score for each GBM patient from TCGA database was obtained from The Cancer Immunome Database (TCIA, https://tcia.at/home). Differential analysis of immunotherapy scores for GBM patients was carried out and visualized by ‘ggpubr’ package.

### Construction and validation of DRGs based prognostic model

Firstly, the RNA-seq data of LGG samples from TCGA database, which were treated as validation cohort, were transformed to transcripts per million (TPM) values and log2-scale transferred for further analysis. Moreover, the merged data were treated as the training cohort. The RNA-seq data involved in the validation cohort and training cohort were corrected batch effect. WGCNA was employed to determine the key module which was correlated with survival and differentiation based on the expression patterns of DRGs in the training cohort. Univariate cox regression analysis was carried out to screen out the genes involved in the key module with prognostic values by using ‘survival’ package, in which *p* < 0.005 was considered as statistically significant. Additionally, the list of transcription factors was downloaded from cistrome cancer database (http://cistrome.org/CistromeCancer/) and co-expression analysis was utilized to investigate the correlation between transcription factors and the prognostic DRGs by using ‘dplyr’ and ‘limma’ packages. Afterwards, the LASSO regression algorithm was employed to further screen out DRGs with prognostic values. The Receiver Operating Characteristic (ROC) curves and Kaplan–Meier analysis were used to evaluate the performance of the prognostic model in which R packages including ‘survival’, ‘glmnet’, ‘survminer’ and ‘timeROC’ were employed. The risk score for each patient was calculated according to the following formula: risk score = $\mathop \sum \limits_{i = 1}^{n} coef{\text{DRG}}i*EXP {\text{DRG}}i$ in which the $coef{\text{DRG}}i$ means the coefficient for the $i$th DRG, and the $EXP {\text{DRG}}i$ represents the expression level of the $i$th DRG in the prognostic model. Univariate and multivariate cox regression analysis were applied to evaluate the prognostic value of risk score. Patients were divided into high-risk group and low-risk group with the cut off of the median risk score. Additionally, another independent cohort from CGGA database (data set ID: mRNAseq_325) was used to further verify the performance of the prognostic model.

### Construction of nomogram

Nomogram combing risk score and clinicopathological factors was introduced to fulfill the prognostic model by using ‘rms’ and ‘regplot’ R package. Predictions for survival at the time of 1-, 2- and 3- years were accomplished. Calibration curves were carried out to explore the accuracy of the nomogram.

### Prediction of immunotherapy response between patients of different groups

An unsupervised subclass mapping method was used to predict the response to anti-PD1 and anti-CTLA4 immunotherapy for patients in different groups (https://cloud.genepattern.org/gp/).

### Prediction of candidate targeted drugs

The differentially expressed genes between the high and low-risk groups in the merged data were determined with |log2 (FC)|> 0.585 and adjusted *p* values < 0.05. The up-regulated genes and down-regulated genes were put into Connectivity Map (CMap) (version build 02) database for identification of candidate small molecule drugs. Then, the drugs with enrichment scores < − 0.800 and *p* values < 0.05 were selected as candidate targeted drugs.

### Statistical analysis

The comparisons across multi-groups were carried out using Kruskal–Wallis test, and the Wilcoxon test was utilized to conduct the comparisons between two groups. The distribution of categorical variables between subgroups was analyzed through Chi-square tests. The student’s t-test was implemented to compare the continuous data between pairs of subgroups. Pearson’s correlation test was employed to investigate the correlations between normally distributed variables, while correlations between non-normally distributed variables were evaluated by Spearman’s correlation test. The log rank test was employed to examine the statistically differences of the overall survival in the Kaplan–Meier analysis. R software in version 4.1.1 (Institute for Statistics and Mathematics, Vienna, Austria) was utilized to accomplish the statistical analysis and visualization of the results (https://www.r-project.org/).

### Ethics declarations

Ethics committee approval for our study was not required because the data were obtained from publicly available databases.

## Supplementary Information

Supplementary Information.

## Data Availability

We declare that the data sets supporting the findings of this study are available in the TCGA database (https://portal.gdc.cancer.gov/), GEO database (https://www.ncbi.nlm.nih. gov/geo/) and CGGA database (Chinese Glioma Genome Atlas, http://cgga.org.cn/index.jsp).
