# Supplementary material for: Single cell RNA sequencing reveals differentiation related genes with drawing implications in predicting prognosis and immunotherapy response in gliomas
Source: Sci Rep. 2022 Feb 3;12:1872. doi: 10.1038/s41598-022-05686-x (PMC8814011; doi:10.1038/s41598-022-05686-x)
Supplement: Supplementary file 1 — Supplementary Information. [file 41598_2022_5686_MOESM1_ESM.pdf]

Single cell RNA sequencing reveals differentiation related genes with drawing implications in predicting prognosis and immunotherapy response in gliomas

Zijian Zhou, JinHong Wei, Zeruo Yang, Yue Bao, Wenbo Jiang, Bin Lu, Weimin Wang, Luo Li

Supplementary table 1. Clinicopathological features of patients in GSE103224

GEO accession	Age	Gender	Location	Diagnosis	IDH1 status
**GSM2758471**	49	Female	right frontal	Glioblastoma, WHO grade IV	mutant type
**GSM2758472**	62	Male	left temporal	Glioblastoma, WHO grade IV	wild type
**GSM2758473**	65	Male	left temporal	Glioblastoma, WHO grade IV	wild type
**GSM2758474**	74	Male	right frontal	Glioblastoma, WHO grade IV	wild type
**GSM2758475**	56	Female	left temporal	Anaplastic Astrocytoma, WHO grade III	wild type
**GSM2758476**	63	Female	left temporal	Glioblastoma, WHO grade IV	wild type
**GSM2758477**	50	Male	left temporal	Glioblastoma, WHO grade IV	wild type
**GSM2940098**	59	Male	right parietal	Glioblastoma, WHO grade IV	wild type

Supplementary table 2. Clinicopathological features of patients with GBMs in TCGA database

Covariates		N(%)
**Gender**	Female	49(35%)
	Male	91(65%)
**Age**	<60	67(47.86%)
	>=60	73(52.14%)
**Disease Free Status**	DiseaseFree	16(11.43%)
	Recurred/Progressed	91(65%)
	unknow	33(23.57%)

**Sample Type**	Primary	127(90.71%)
	Recurrence	13(9.29%)
**Neoplasm Status**	Tumor free	12(8.57%)
	With tumor	113(80.71%)
	unknow	15(10.71%)
**History of LGG**	N0	135(96.43%)
	Yes	5(3.57%)
**Karnofsky Performance Score**	<80	27(19.29%)
	>=80	82(58.57%)
	unknow	31(22.14%)

Supplementary table 3. Clinicopathological features of patients with LGGs in TCGA database

**Covariates**		**N(%)**
**Gender**	Female	224(44.53%)
	Male	279(55.47%)
**Age**	<=60	443(88.07%)
	>60	60(11.93%)
**Disease Free Status**	DiseaseFree	306(60.83%)
	Recurred/Progressed	162(32.21%)
	unknow	35(6.96%)
**Grade**	G2	242(48.11%)
	G3	260(51.69%)
	unknow	1(0.2%)
**Histologic Type**	Astrocytoma	189(37.57%)
	Oligoastrocytoma	128(25.45%)
	Oligodendroglioma	186(36.98%)
**IDH1 Mutation**	NO	32(6.36%)
	YES	91(18.09%)
	unknow	380(75.55%)
**Postoperative radiotherapy**	NO	118(23.46%)
	YES	141(28.03%)
	unknow	244(48.51%)
**Therapy Outcome**	Complete Remission/Response	83(16.5%)
	Partial Remission/Response	50(9.94%)
	Progressive Disease	36(7.16%)
	Stable Disease	59(11.73%)
	unknow	275(54.67%)

Supplementary table 4. Clinicopathological features of patients in GSE4271

**Covariates**		**N(%)**
**Gender**	Female	24(32%)

	Male	51(68%)
**Age**	<60	67(89.33%)
	>=60	8(10.67%)
**Grade**	G3	21(28%)
	G4	54(72%)

Supplementary table 5. Clinicopathological features of patients in GSE43378

**Covariates**		**N(%)**
**Gender**	Female	14(29.17%)
	Male	34(70.83%)
**Age**	<60	27(56.25%)
	>=60	21(43.75%)
**histological type**	Anaplastic astrocytoma	7(14.58%)
	Anaplastic oligoastrocytoma	2(4.17%)
	Anaplastic oligodendroglioma	4(8.33%)
	Astrocytoma	5(10.42%)
	GBM	30(62.5%)
**Grade**	G2	5(10.42%)
	G3	13(27.08%)
	G4	30(62.5%)

Supplementary table 6. Clinicopathological features of patients in the validation cohort from CGGA database (data set ID: mRNAseq_325)

**Covariates**		**N(%)**
**Recurrent type**	Primary	222(71.84%)
	Recurrent	58(18.77%)
	Secondary	29(9.39%)
**Grade**	G2	98(31.72%)
	G3	74(23.95%)
	G4	137(44.34%)
**Gender**	Female	116(37.06%)
	Male	197(62.94%)
**Age**	<60	280(89.46%)
	>=60	33(10.54%)
**Radiotherapy status**	treated	241(79.54%)
	untreated	62(20.46%)
**Chemotherapy status**	treated	190(63.33%)
	untreated	110(36.67%)
**IDH mutation status**	Mutant	167(53.53%)
	Wildtype	145(46.47%)
**1p19q codeletion status**	Codel	62(20.33%)
	Non-codel	243(79.67%)
**MGMTp methylation status**	methylated	152(51.53%)
	un-methylated	143(48.47%)

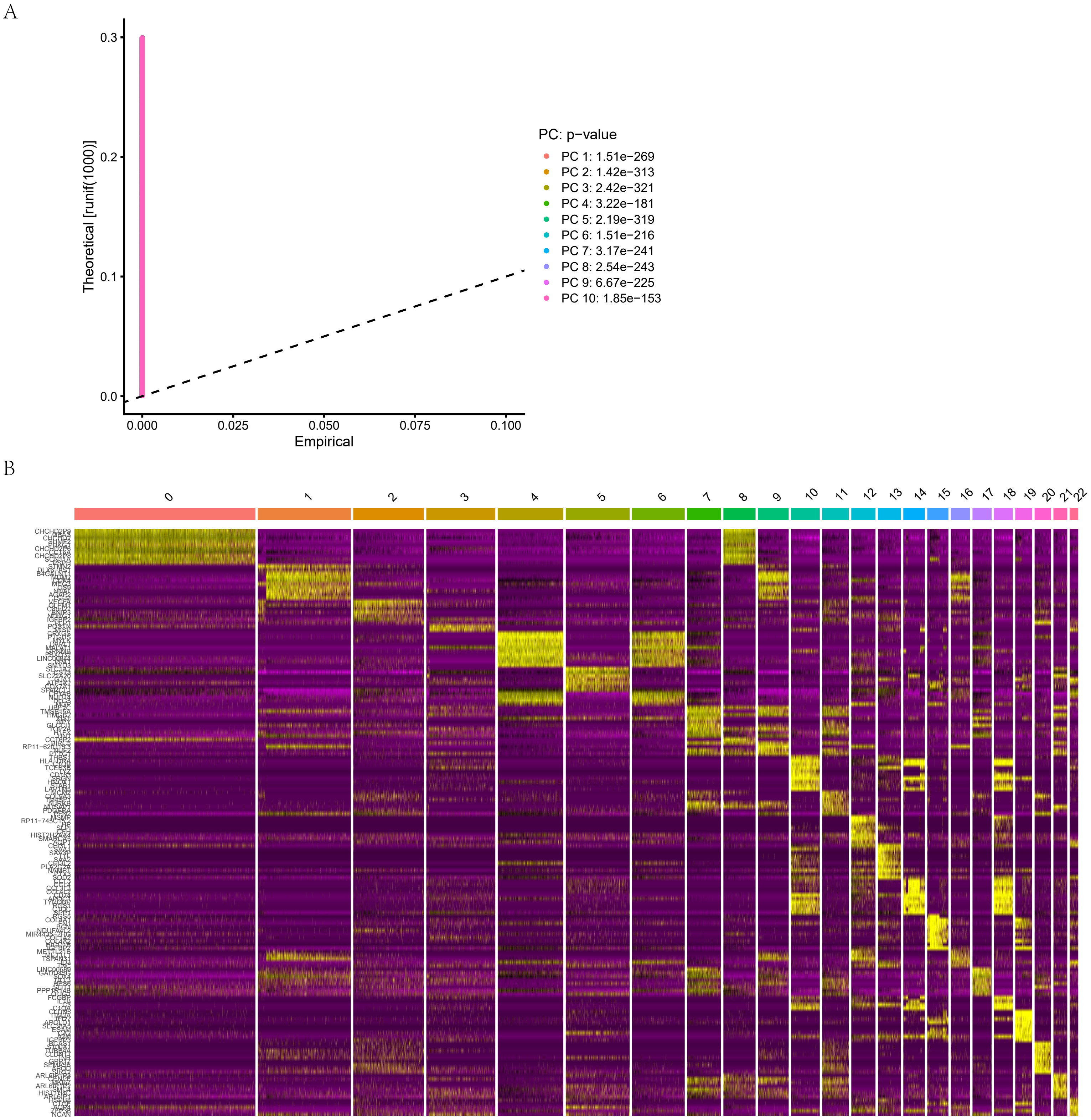

FigS1. (A) Ten PCs with significant differences were identified. (B)Heatmap showing the expression patterns of top 10 differentially expressed marker genes for each cluster. PCs: principal components.

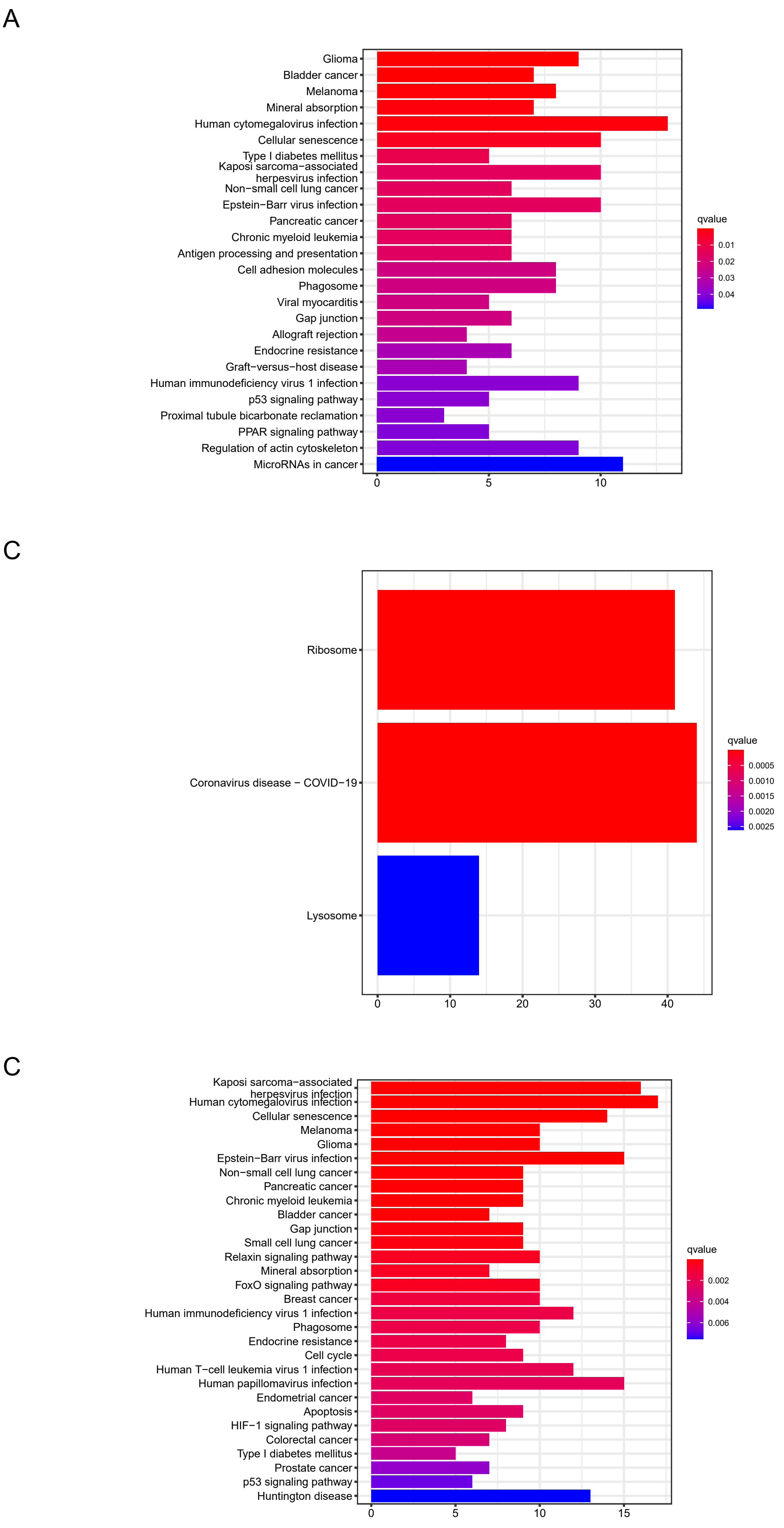

FigS2. Functional annotation for three differentiation states. (A-C) KEGG pathway enrichment analysis of marker genes for state 1-3. KEGG: Kyoto Encyclopedia of Genes and Genomes. That data from the Kanehisa laboratory were used for the functional annotation (www.kegg.jp/kegg/kegg1.html).

A

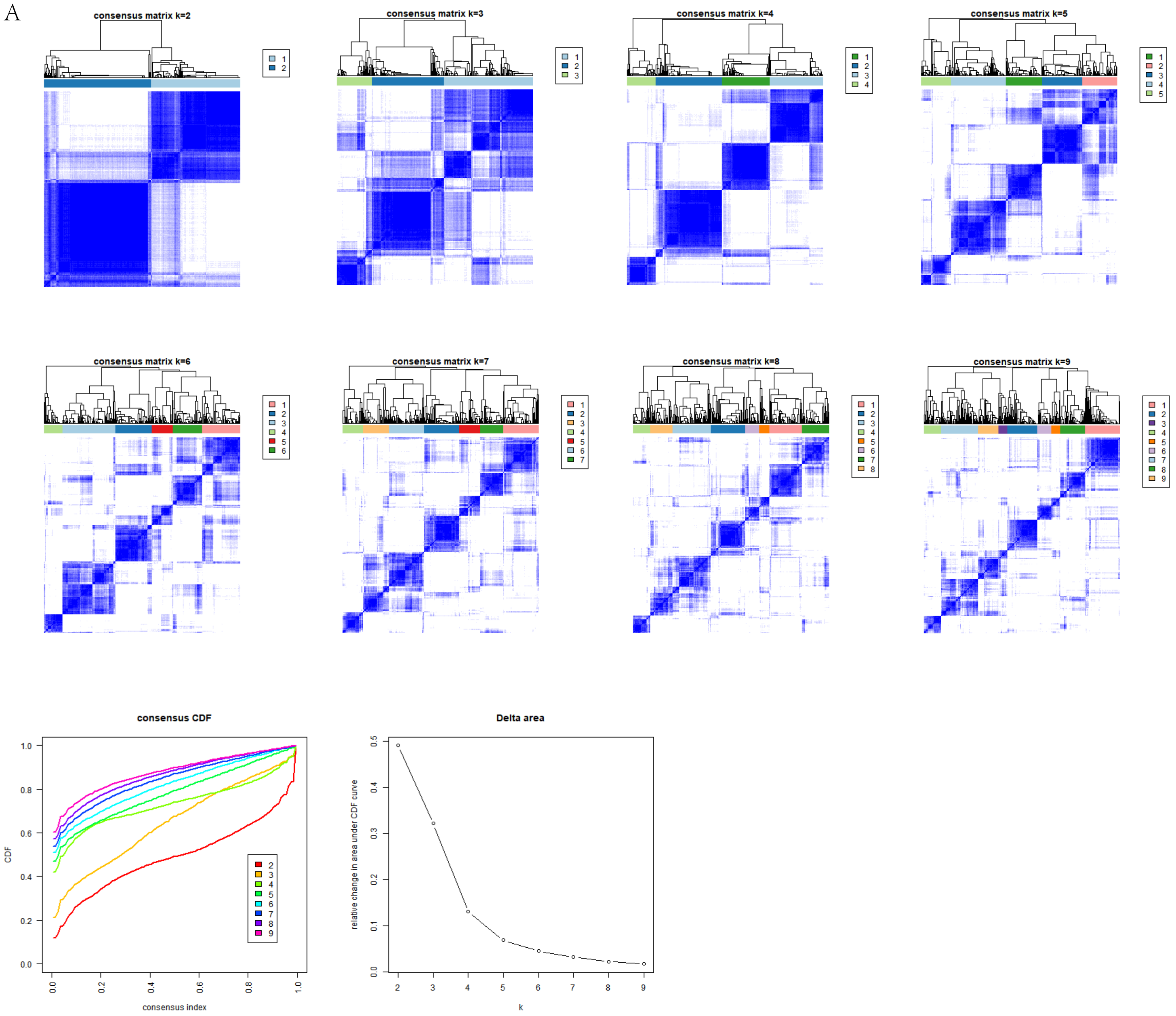

B

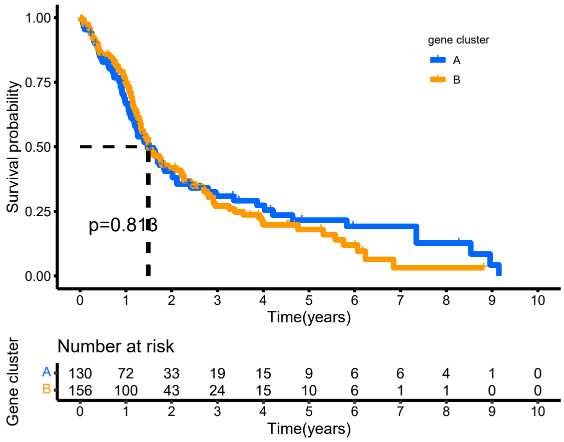

FigS3. (A) The results of consensus clustering analysis for glioma patients based on highly variable genes identified in single-cell RNA sequencing data. (B) Overall survival analysis between patients in the two clusters.

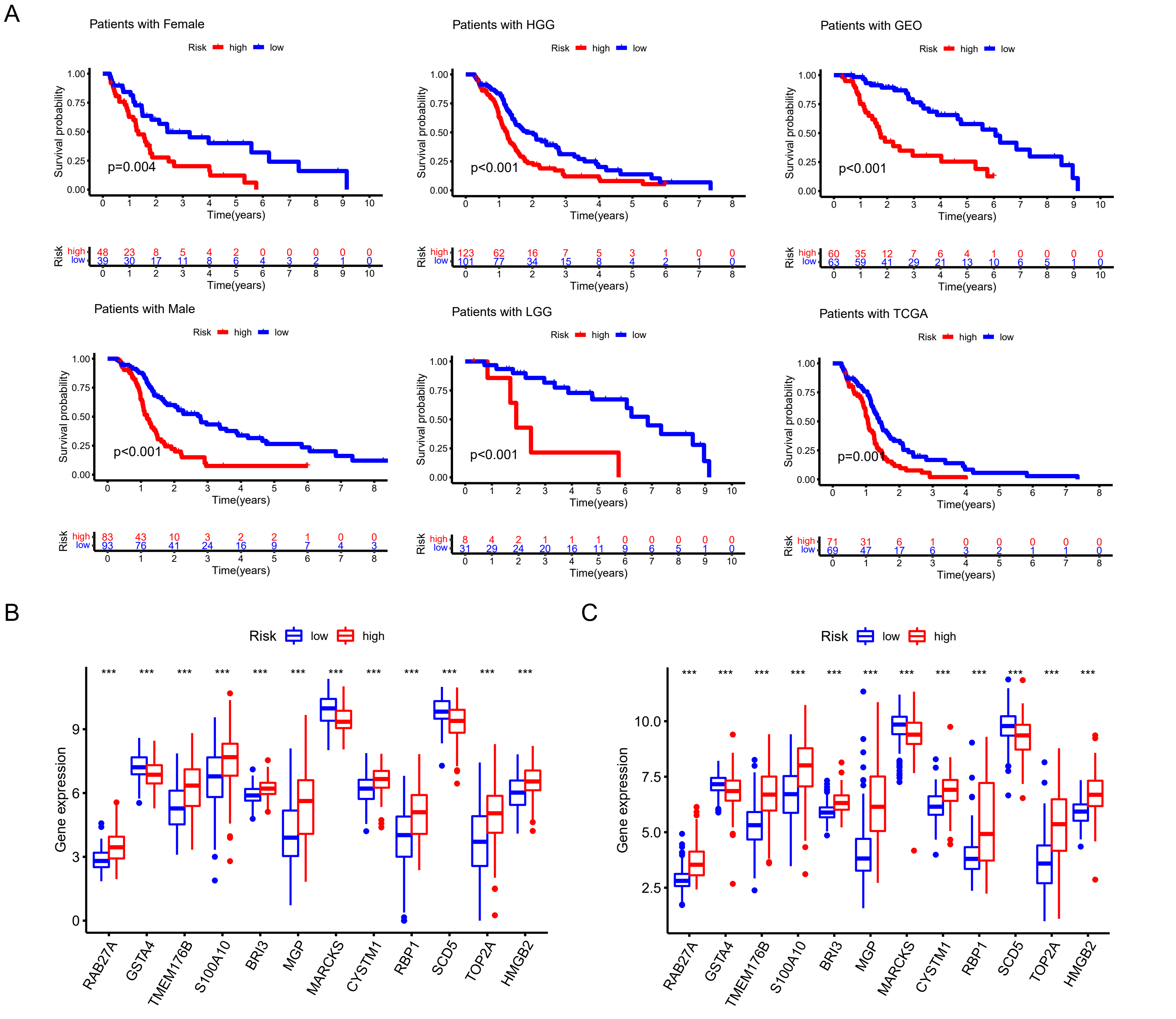

FigS4. (A) Kaplan-Meier analysis between the low-risk and high-risk groups with different clinicopathological features. (B, C) Comparisons of the expression patterns of the 12 prognostic genes in our model between high-risk and low-risk groups in the training cohort (B) and validation cohort (C), respectively. * means P < 0.05, ** means P < 0.01, and ***means P <0.001

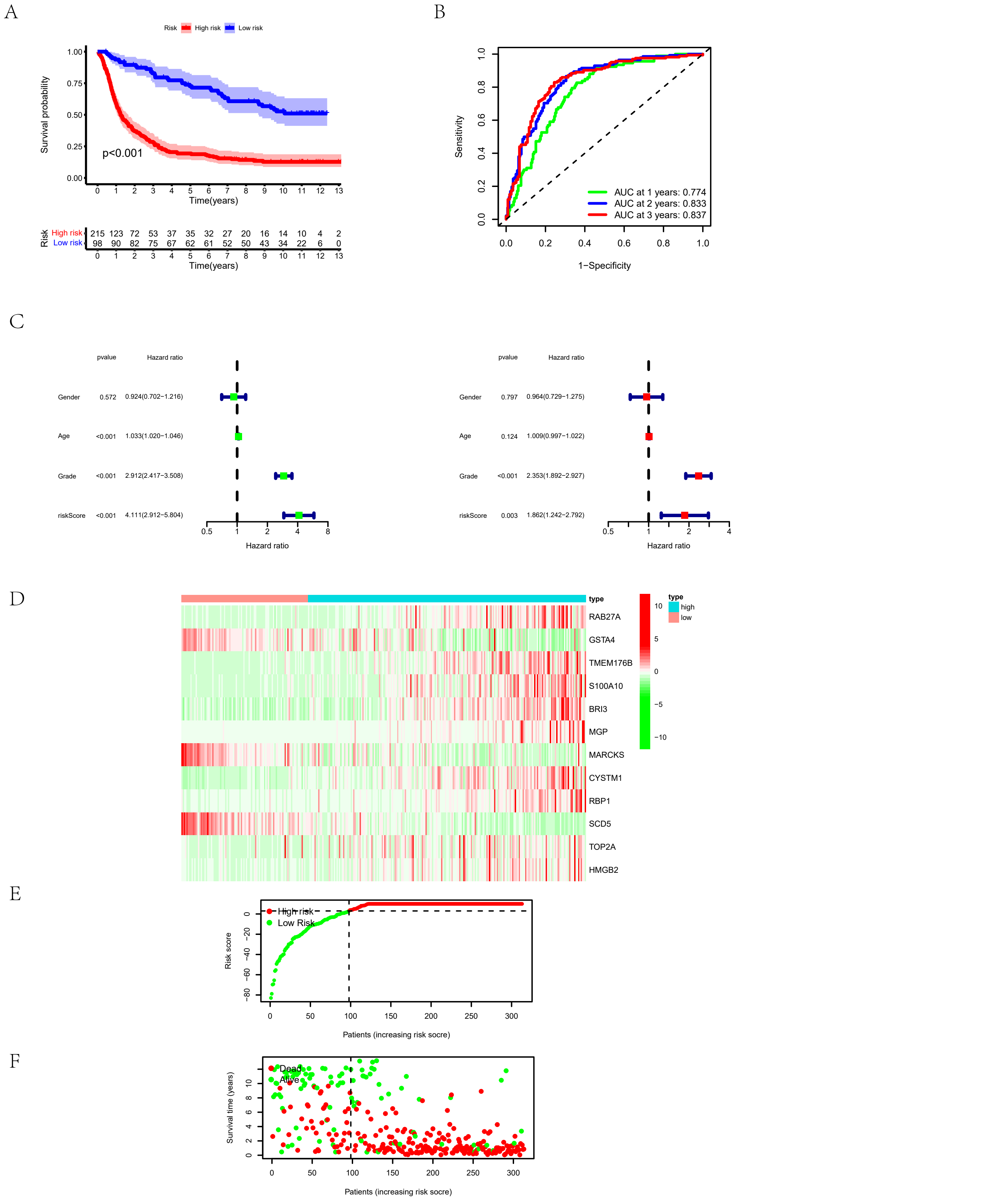

FigS5. Validation of the prognostic model in an independent cohort (data set ID: mRNAseq_325). (A) Kaplan-Meier survival analysis between the low-risk group and the high-risk group. (B) The ROC curves for predicting 1-year, 2-year and 3-year overall survival. (C) Forest plot showing the results of univariate (left panel) and multivariate (right panel) cox regression analysis of the risk score and clinicopathological features. (D-F) Heatmap showing the expression patterns of prognostic genes in the model (D), the curve of risk scores (E) and scatter plot of survival states (F).

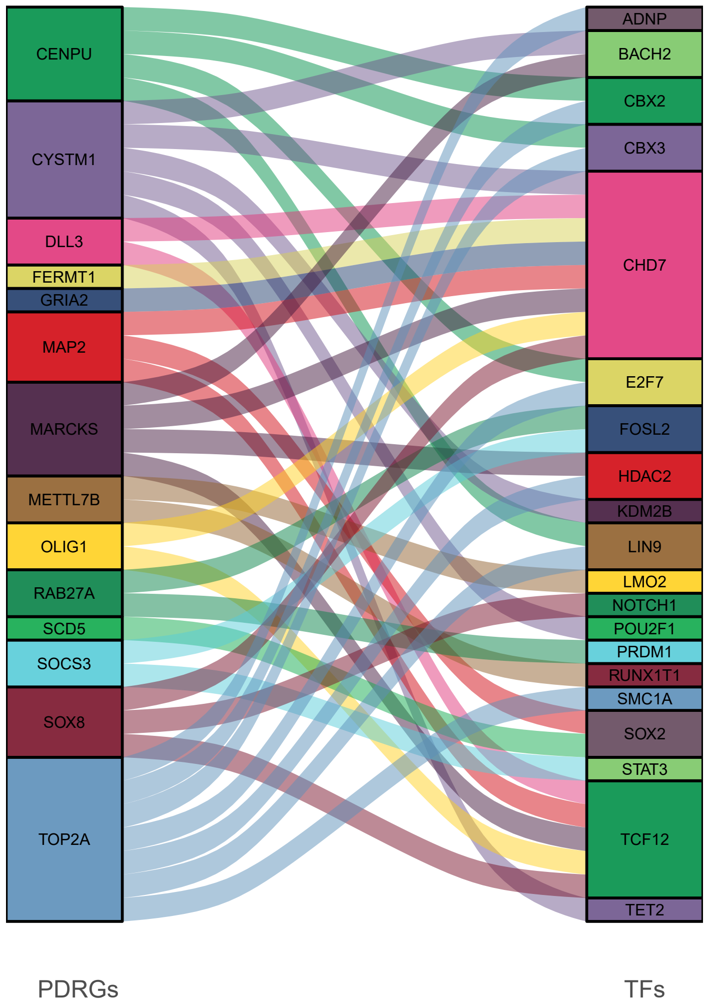

FigS6. Alluvial diagram showing the correlation between prognostic DRGs and the corresponding transcriptional factors. PDRGs: prognostic differentiation related genes, TFs: transcriptional factors
